# Case Report: A case of Tatton-Brown–Rahman syndrome featuring mitral annular disjunction and mitral valve prolapse due to a novel mutation site in the *DNMT3A* gene

**DOI:** 10.3389/fcvm.2024.1507318

**Published:** 2025-01-20

**Authors:** Zhong-jiao Xu, Ru-ming Shen, Wu-ming Hu, Lin-chun Lv, Zhen-hua Shi, Li Lin

**Affiliations:** Department of Cardiology, The Fifth Affiliated Hospital of Wenzhou Medical University, Lishui Central Hospital, Zhejiang, China

**Keywords:** TBRS, Tatton-Brown–Rahman syndrome, *DNMT3A*, mitral valve prolapse, mitral annular disjunction

## Abstract

A 13-year-old child presented with specific facial features, overgrowth, and intellectual disability. Echocardiography revealed the presence of a large pericardial effusion, left ventricular enlargement, mitral annular separation, and mitral valve prolapse with moderate regurgitation. These symptoms suggested a possible genetic disorder. High-throughput sequencing revealed a specific mutation in the *DNMT3A* gene (NM_175629.2:c.2408 + 1G > A) associated with Tatton-Brown–Rahman syndrome. The patient's condition was alleviated through accurate diagnosis and comprehensive treatment measures, including psychological and social support. Regular follow-ups to monitor the disease's progress and the effectiveness of treatment, along with timely adjustments to the treatment plan, can not only effectively reduce the child's symptoms and improve their quality of life but may also help prevent the potential risk of sudden death.

## Introduction

Tatton-Brown–Rahman syndrome (TBRS), also known as *DNMT3A* overgrowth syndrome, was first characterized by Tatton-Brown and colleagues in 2014 following the identification of the *DNMT3A* gene. This multisystemic condition affects multiple bodily systems, including the nervous system, musculature, and vasculature, and is often associated with a spectrum of intellectual disabilities and distinctive facial features. This article presents a rare case of TBRS attributable to a novel mutation in the *DNMT3A* gene, which is characterized by mitral annular separation with associated mitral valve prolapse (MVP) and regurgitation, a cardiac phenotype seldom observed in TBRS. We conducted an in-depth analysis of the case and examined its clinical features, potential outcomes, and therapeutic strategies. The aim is to increase clinical awareness, refine comprehensive treatment approaches, and guide the long-term management of this rare disorder, thereby improving the quality of life for individuals with TBRS and decreasing the risk of sudden death.

## Case presentation

The child, a 13-year-old boy, visited the Department of Pediatrics at Lishui Central Hospital in June 2022, complaining of “chest tightness and abdominal pain secondary to activity ongoing for half a year.” The parents were healthy, had normal intelligence, and were not close relatives. The child had a 5-year-old younger brother who had an overall healthy body. There was no family history of genetic metabolic diseases. The child was born when his parents were 27 and 24 years old, and the mother had a normal pregnancy based on examination. She denied a history of exposure to radioactive or harmful substances, and she confirmed a full-term cesarean section with no history of birth injury or asphyxia. At birth, the child had a length of 55 cm, a weight of 5.0 kg, and a head circumference of 40 cm. The mother confirmed the child's ability to look up within 3 months, sit up within 6 months, and walk within 12 months. No medical complications were reported in the first year of life. The patient's intelligence was found to be below normal until the age of 10, with a Wechsler Intelligence Scale for Children -Fourth Edition score (WISC-IV) score of 80, high myopia, bilateral visual acuity of 750°, flat-footedness, and vocal cord paralysis.

Furthermore, 6 months prior to admission, the child experienced an onset of chest tightness following physical activity, reduced exercise tolerance, and paroxysmal dull pain in the middle and upper abdomen. He was admitted to the pediatrics department of our hospital in June 2022. On admission, a physical examination revealed the following: temporal temperature of 37.1°C, heart rate of 63 beats per minute, blood pressure of 119/67 mmHg, height of 182 cm, weight of 73 kg, distended jugular veins, no superficial lymph node enlargement, diminished breath sounds in both lower lungs, slightly moist rales, a pronounced second heart sound on auscultation, a flat and soft abdomen, no abdominal tenderness, absence of rebound pain, no muscle guarding, and no rigidity or percussion pain in the kidney and liver regions. No edema was observed in the lower limbs or arachnoid toes. Auxiliary examinations, including routine blood tests, erythrocyte sedimentation rate (ESR), and B-type natriuretic peptide (BNP) analysis, revealed no significant abnormalities. Chest and abdominal CT scans revealed a small amount of pleural and abdominal effusion. Echocardiography revealed a large pericardial effusion, with the thickest part measuring 4.0 cm. Consequently, the clinical physician promptly performed pericardial percutaneous catheter drainage. The aspirated fluid was reddish in color, and laboratory tests identified it as a bloody exudate. A cytological analysis reported that the fluid consisted of 68% neutrophils, 20% lymphocytes, 10% macrophages, and 2% eosinophils. The smear showed a predominance of nucleated cells, primarily neutrophils, with no detectable malignant cells. Analysis of the pericardial effusion revealed the following: total protein, 49.1 g/L; glucose, 2.81 mmol/L; lactate dehydrogenase, 527 U/L; Rivalta test, positive; acid-fast bacteria stain, negative for acid-fast bacilli; and tuberculosis DNA assay, negative. The etiology of the pericardial effusion remains unclear.

**Figure 1:**
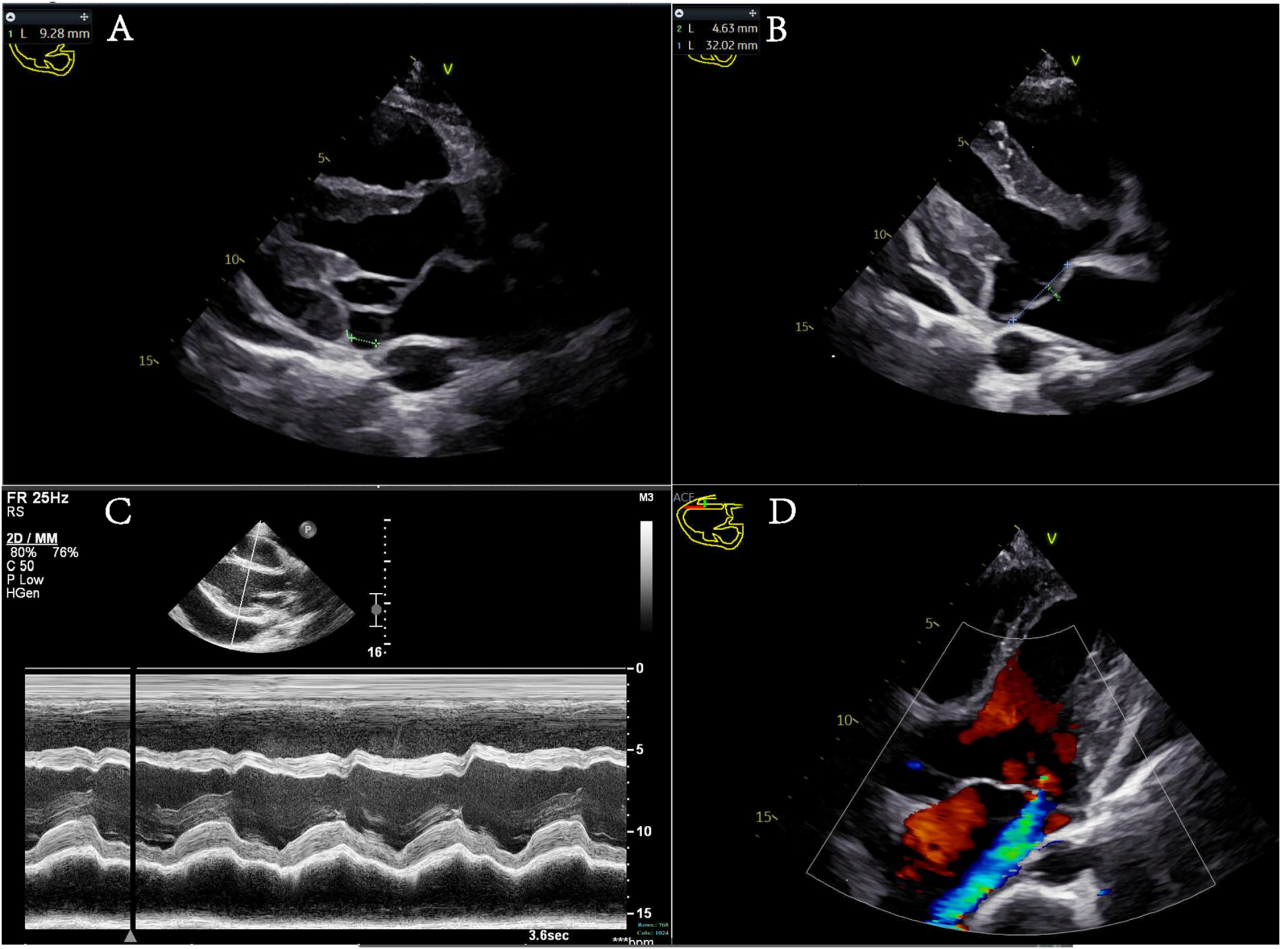
**(A–C)** Different views of the parasternal long-axis section of the left ventricle. **(A)** Mitral annular disjunction during systole, with a green dashed line indicating the separation distance of 9.28 mm. **(B)** The anterior leaflet of the mitral valve protrudes into the left atrium during systole, with a protrusion distance of 4.63 mm. **(C)** An M-mode echocardiogram displaying the ventricular wave complex. Left ventricular systolic function is normal, with signs of a pericardial effusion. **(D)** The apical five-chamber view showing mitral valve prolapse with moderate regurgitation.

The day after admission, the patient and his family, who were concerned about the child's condition, requested a transfer to Wenzhou Yuying Children's Hospital for treatment. Upon admission, echocardiography revealed anterior MVP with moderate regurgitation and a pericardial effusion. The diagnoses included a pericardial effusion, heart failure, pneumonia, a bilateral pleural effusion, vocal cord paralysis, and a history of tonsillectomy. After a pleural puncture and anti-infection and symptomatic treatment, pleural effusion samples were sent for Pathogen Metagenomics Sequencing (PM-seq), a high-throughput genetic detection method for pathogenic microorganisms. The results revealed that no pathogenic microorganisms, including bacteria, viruses, fungi, parasites, *Mycobacterium tuberculosis* complex, mycoplasma, chlamydia, or *Rickettsia*, were detected. Following symptomatic treatment, the patient's symptoms improved, leading to discharge from the hospital. The patient continued to experience chest tightness after activity after discharge, however, and was transferred to our hospital's emergency department. Further auxiliary examinations were conducted, including routine blood tests and measurements of blood coagulation, liver function, albumin, renal function, electrolytes, fasting blood glucose, fecal routine, tumor markers, thyroid function, ESR, BNP, hepatitis B triple system, serum protein electrophoresis, antinuclear antibody, antineutrophil cytoplasmic antibody, growth hormone, glomerular basement membrane, specific proteins, antistreptolysin O (ASO), rheumatoid factor, HIV, and syphilis, all of which were negative. A plain chest scan revealed a few fibrous foci in both lungs. Brain MRI with plain scan and diffusion imaging revealed no obvious abnormalities. An electroencephalogram (EEG) revealed mild abnormalities. Echocardiographic monitoring by a cardiac specialist (Figure 1) revealed left ventricular enlargement, mitral annular disjunction (MAD) (separation distance: 9.28 mm), anterior MVP with moderate regurgitation, and a small amount of pericardial effusion. Aortic computed tomography angiography (CTA) (from the mandibular angle to the femoral neck) revealed a widening of the aortic root, enlargement of the heart, and a small pericardial effusion. A large number of causes of pericardial and pleural effusions were investigated, excluding common causes such as malnutrition, thyroid dysfunction, hepatic and renal dysfunction, tumors, and tuberculosis.

Given the patient's distinctive symptoms, which included tall stature, gynecomastia, intellectual disability, ptosis, high myopia, vocal cord paralysis, long limbs, flat feet, arachnodactyly, massive pericardial effusion, aortic root dilatation, mitral annular disjunction, and MVP, an underlying genetic disorder was considered. With the consent of the family, 2 ml of peripheral blood was collected from the child and both parents and sent to the Chinese Genomic Data Bank for whole-exome sequencing (WES) and mitochondrial genome sequencing. High-throughput sequencing was performed on the child and the parents, revealing a *DNMT3A*; NM_175629.2: c.2408 + 1G > A mutation in the child. Sanger sequencing was used to verify these findings in the child and the parents. No pathogenic mutations were identified in the parents. This mutation is located at a canonical splice site and has not been previously reported in the literature.

The patient received standardized treatment by specialists, which included pericardiocentesis catheter drainage, diuretic therapy to improve the cardiac load, anti-infection measures, and other comprehensive treatments. As a result, the patient's symptoms of chest tightness and shortness of breath were alleviated. Currently, the patient is undergoing regular treatment in the Children's Health Care Department, where they are receiving comprehensive training in intelligence, behavior, and social communication. In addition, the patient is engaged in regular follow-up care aimed at improving the prognosis and preventing the risk of sudden death. The treatment time nodes are shown in the Figure 3.

## Discussion

TBRS (OMIM 615879), also known as *DNMT3A* overgrowth syndrome, was first reported by Tatton-Brown et al. (1, 2) with the related gene *DNMT3A* in 2014; this is a relatively newly identified congenital overgrowth syndrome. TBRS (2) is a complex multisystem disease involving many different tissues, including the nervous system, muscles, and blood. The most common clinical manifestations include intellectual disability (ranging from mild to severe), overgrowth (defined as above-average height and/or head circumference greater than 2 standard deviations above the mean), distinctive facial features (including low-set, heavy, horizontal eyebrows and prominent upper central incisors, which may become more pronounced with age), joint hypermobility, hypotonia, behavioral/mental disorders (most common in autism spectrum disorders), scoliosis, and non-febrile convulsions. These characteristics can be subtle but are distinctive for individuals with TBRS. In addition, several types of cardiac malformations, including atrial septal defects, mitral and tricuspid regurgitation, patent ductus arteriosus, hypertrophy of the aortic root, and atrioventricular reentrant tachycardia, have been reported in individuals with TBRS. Notably, two individuals with cardiac malformations, specifically patent ductus arteriosus (COG1961 and COG2006) were identical twins. The deletion of the entire *DNMT3A* gene, which is associated with TBRS, affects more than 40 genes. Therefore, the patent ductus arteriosus in these individuals may be attributed to their twin status, alternative genes in the deletion region, or the combined effect of many deleted genes (2).

**Figure 2:**
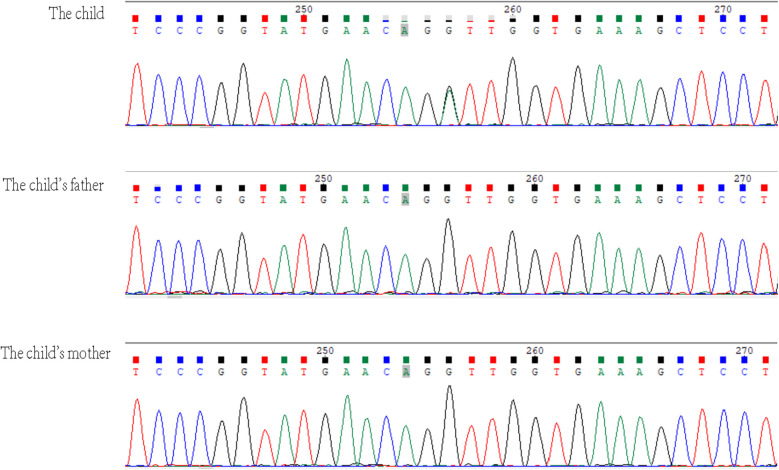
In the *DNMT3A* gene, the child had a heterozygous variation NM_175629.2:c.2408 + 1G > A, which is not present in the parents. The arrow indicates the mutation site.

TBRS is inherited in an autosomal dominant manner. The DNA methyltransferase 3*α* (DNMT3A) protein is involved in the *de novo* methylation of DNA, a process essential for genome regulation and development. TBRS is caused by abnormal methylation due to pathogenic germline variations in the *DNMT3A* gene, which leads to gene regulation dysfunction and related clinical symptoms (3, 4). Initially, somatic mutations in *DNMT3A* were identified in acute myeloid leukemia (5). TBRS is typically associated with *de novo* mutations. A variety of pathogenic mutations in the *DNMT3A* gene, including missense, non-sense, frameshift, splicing, in-frame deletions, and whole-gene deletions, have been reported in previous studies on TBRS, with some cases involving identical twins (1, 2, 6–10). In TBRS patients, the majority of reported pathogenic variants in the *DNMT3A* gene are novel, although some are familial (11). The patient in this case report has a heterozygous variation in the *DNMT3A* gene, NM_175629.2:c.2408 + 1G > A. Sanger sequencing was used to verify mutations in the patient and both parents (Figure 2), and no pathogenic mutations were found in the parents. The mutation site is located at a canonical splice site and this specific mutation has not been previously reported. According to the ClinVar database, a *DNMT3A* loss-of-function variant is a known pathogenic mechanism for TBRS and so this novel mutation is associated with very strong evidence of pathogenicity based on PVS1 (very strong evidence of pathogenicity). TBRS is considered to exhibit autosomal dominant inheritance, and the patient, who is a heterozygous carrier, fits this model. However, the genetic origin of this mutation is unclear; neither parent exhibits the phenotype or carries the mutation, and the variant site has not been reported in reference population databases, suggesting a novel mutation. The supporting evidence for pathogenicity (PM2) aligns with the ACMG guidelines, which classify the mutation as a suspected pathogenic mutation based on PVS1 + PM2. Combined with the above evidence, this genetic site is likely pathogenic, leading to the diagnosis of TBRS.

**Figure 3:**
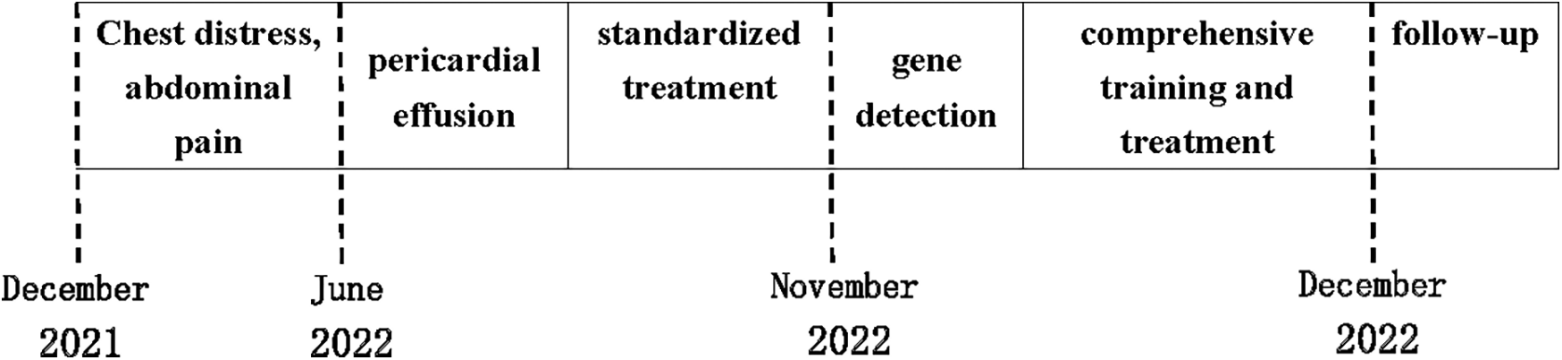
The treatment time nodes.

The child's primary clinical manifestations included chest tightness and decreased exercise endurance following physical activity. Echocardiography revealed a large pericardial effusion and mitral annular disjunction. The separation was characterized by anterior MVP with moderate regurgitation and aortic CTA indicated a widening of the aortic root. Aortic root dilatation has been previously reported in several patients with TBRS (2, 12, 13). This child's enlarged aortic root is consistent with previously reported cases. Initially, a large bloody pericardial effusion was suspected to be due to hepatorenal dysfunction, thyroid dysfunction, malnutrition, pathogen infection, tumor, tuberculosis, trauma, or heart rupture, but these causes were subsequently excluded. The cause was instead attributed to the local rupture of cardiac small vessels during the overgrowth process and chronic exudation, leading to a bloody pericardial effusion. During a follow-up visit, only 3 mm of pericardial effusion was found. No reports on children with TBRS complicated by a pericardial effusion currently exist, and there are no relevant literature reports on mitral annular disjunction and anterior MVP in patients with TBRS. MVP (14) is characterized by the thinning, lengthening, and/or rupture of chordae tendineae, typically due to elastic fiber deficiency, and is often associated with prolapse and mitral regurgitation of varying severities. MVP is generally considered sporadic but can also be a common pathway for various genetic and acquired diseases (e.g., Marfan syndrome, Ehlers–Danlos syndrome, aneuploidy syndrome, Loeys–Dietz syndrome, or pseudoxanthoma elasticum), which may weaken the connective tissue of the valve, leading to valvular elongation, thickening, and degeneration (15). A study by Dejgaard et al. (14) revealed that the incidence of ventricular arrhythmias in patients with MAD is high and not associated with MVP, suggesting an increased risk of malignant arrhythmias in patients with mitral annular disjunction. Research by Aabel et al. (16) demonstrated that MAD itself is an arrhythmogenic disease, with predictors of the first severe arrhythmia including frequent ventricular extrasystole, non-persistent ventricular tachycardia, increased left ventricular diameter, and a large separation distance of the posterolateral mitral annulus. At present, there are no specific guidelines for the diagnosis or treatment of MAD. Regular dynamic electrocardiograms (ECG) monitoring is recommended to detect potential malignant arrhythmias. There is a scarcity of data and clinical trials regarding the selection of patients with arrhythmogenic mitral valve syndrome for primary prophylactic Implantable Cardioverter-Defibrillator (ICD) implantation, and the incidence of secondary prophylactic ICD is even greater (16). Therefore, no relevant data suggest that ICD implantation can significantly benefit these patients. The proposed mechanism of arrhythmia syndrome suggests that (17) mitral annulus repair surgery for mitral annular separation and MVP may play a role in limiting the risk of further arrhythmia. According to the relevant data, mitral annular disjunction in patients with TBRS may be attributed to the overgrowth of tissues and organs caused by TBRS-related *DNMT3A* gene mutations, which promote the proliferation of the stem cell/progenitor cell pool, leading to an excessive increase in the number of cells during organ morphological differentiation and growth. Research on the mechanism of this type of disease is scarce, and the specific causal mechanism needs to be clarified by more rigorous basic and clinical research.

The patient in this case report is the first case in which TBRS is complicated with a massive pericardial effusion caused by a novel mutation in the *DNMT3A* gene, within both the Chinese and international populations. The mitral annulus is isolated by MVP.

The heterozygous variant in the *DNMT3A* gene c.2408 + 1G > A is a novel mutation. The patient presented with a range of clinical manifestations, including tall stature, long limbs, gynecomastia, intellectual disability, ptosis, high myopia, vocal cord paralysis, flat feet, arachnodactyly (a spider-like elongation of fingers and toes), and a large pericardial effusion. In addition, there were specific cardiac manifestations, such as aortic root dilatation, mitral annular disjunction, and mitral anterior leaflet prolapse. The clinical manifestations of TBRS overlap with those of other syndromes characterized by overgrowth and intellectual disability (10), including Heyn–Sproul–Jackson syndrome (OMIM:618724), Ehlers–Danlos syndrome (OMIM:617821), and Malan syndrome (OMIM:614753). TBRS can be associated with other major clinical manifestations in patients and can be distinguished by disease-specific symptoms, including facial features, overgrowth, and intellectual disability syndrome. When clinical symptoms are indistinguishable, further diagnostic assistance can be sought through full exome analysis, microarray comparative genomic hybridization, and single-nucleotide polymorphism (SNP) microarrays to facilitate disease diagnosis. Diseases associated with cardiac structural changes, such as those mentioned above, are prone to complications such as pericardial effusion, malignant arrhythmias, and even sudden cardiac death. Therefore, it is recommended that dynamic ECGs be conducted and echocardiographic monitoring be intensified during follow-up.

## Conclusion

TBRS is a rare genetic disorder that is seldom associated with congenital heart malformations. Through precise diagnostic measures and personalized therapeutic interventions, along with a comprehensive training program that addresses cognitive, behavioral, and social skills, patients can achieve significant symptom relief, an enhanced quality of life, and better societal integration. Consistent follow-up care and treatment are essential for optimizing patient prognoses and reducing the risk of sudden death in children. This integrated management strategy not only addresses the physical health of patients but also includes psychological and social support, offering a well-rounded approach to caring for individuals with TBRS.

## Data Availability

The original contributions presented in the study are included in the article/Supplementary Material, further inquiries can be directed to the corresponding author.
